# King Cobra and snakebite envenomation: on the natural history, human-snake relationship and medical importance of *Ophiophagus hannah*

**DOI:** 10.1590/1678-9199-JVATITD-2021-0051

**Published:** 2022-01-05

**Authors:** Choo Hock Tan, Aymeric Bourges, Kae Yi Tan

**Affiliations:** 1 Venom Research and Toxicology Laboratory, Department of Pharmacology, Faculty of Medicine, University of Malaya, 50603 Kuala Lumpur, Malaysia.; 2 Université Libre de Bruxelles, 1050 Bruxelles, Belgium.; 3 Protein and Interactomics Laboratory, Department of Molecular Medicine, Faculty of Medicine, University of Malaya, 50603 Kuala Lumpur, Malaysia.

**Keywords:** Proteomics, Venomics, Antivenom, Neutralization, Neglected tropical disease

## Abstract

King Cobra (*Ophiophagus hannah*) has a significant place in many cultures, and is a medically important venomous snake in the world. Envenomation by this snake is highly lethal, manifested mainly by neurotoxicity and local tissue damage. King Cobra may be part of a larger species complex, and is widely distributed across Southeast Asia, southern China, northern and eastern regions as well as the Western Ghats of India, indicating potential geographical variation in venom composition. There is, however, only one species-specific King Cobra antivenom available worldwide that is produced in Thailand, using venom from the snake of Thai origin. Issues relating to the management of King Cobra envenomation (*e.g.*, variation in the composition and toxicity of the venom, limited availability and efficacy of antivenom), and challenges faced in the research of venom (in particular proteomics), are rarely addressed. This article reviews the natural history and sociocultural importance of King Cobra, cases of snakebite envenomation caused by this species, current practice of management (preclinical and clinical), and major toxinological studies of the venom with a focus on venom proteomics, toxicity and neutralization. Unfortunately, epidemiological data of King Cobra bite is scarce, and venom proteomes reported in various studies revealed marked discrepancies in details. Challenges, such as inconsistency in snake venom sampling, varying methodology of proteomic analysis, lack of mechanistic and antivenomic studies, and controversy surrounding antivenom use in treating King Cobra envenomation are herein discussed. Future directions are proposed, including the effort to establish a standard, comprehensive Pan-Asian proteomic database of King Cobra venom, from which the venom variation can be determined. Research should be undertaken to characterize the toxin antigenicity, and to develop an antivenom with improved efficacy and wider geographical utility. The endeavors are aligned with the WHO´s roadmap that aims to reduce the disease burden of snakebite by 50% before 2030.

## Introduction

Snakebite envenomation is a medical emergency characterized by the toxic manifestation of snake venom. It is a major public health problem, a common environmental and occupational hazard affecting many impoverished and geopolitically marginalized populations in the tropics and subtropics [1]. Close to 5 million snakebites occur yearly, resulting in 1.8-2.7 million cases of envenomation. Approximately 81,000-138,000 envenomed victims die each year, while about three times as many survivors continue to suffer various permanent physical disabilities and psychological distress [2,3]. The highest incidence and mortality rates are recorded in South Asia, Southeast Asia and Sub-Saharan Africa, reflecting a combination of factors associated with the developing status, high population density, and venomous snake abundance in the regions [4-6]. The exact extent of the disease burden of snakebite is largely underestimated due to scarce epidemiological data. Climate changes, increased trading of venomous snakes, and the adaptation of venomous species to anthropogenic-modified environments, have also indirectly increased the frequency of contact and conflict between humans and snakes [7,8].

In the past, snakebite envenomation received little attention globally despite its dramatic socio-economic impact on the poor [5,9,10]. It was until 2017 that the World Health Organization (WHO) formally reinstated snakebite envenomation as a priority neglected tropical Disease [11]. Several strategies have since been put forth, with the aim to halve the disease burden of snakebite envenomation by 2030. In this context, South Asia and Southeast Asia are homes to abundant venomous snakes, and are indeed hotspots of snakebite envenomation for solution. Each medically important species has its unique venom composition, and the diversity of venom modulates the pharmacological activities, resulting in varying toxic manifestations and effectiveness of antivenom treatment [12]. Moreover, intra-species variation in snake venom is widely recognized, adding complexity to the management of snakebite as antivenom products are usually manufactured using snake venom sourced from certain locale without considering the potential in-space (geographical) and in-time (ontogenic) variability of venom composition, as well as composition variation possibly caused by sexual dimorphism of the snake [13,14]. This remains true for the case of King Cobra (*Ophiophagus hannah*), a medically important elapid that has a wide biogeographical distribution in Asia. This article reviews the current status of King Cobra from the biological and medical perspectives, highlighting the impact of King Cobra bite on the community and the need for proper management. Recent advances, controversies and challenges in the proteomics, toxicity and neutralization studies of King Cobra venom are discussed. 

## Distribution, taxonomy and behavior

**Figure 1.**
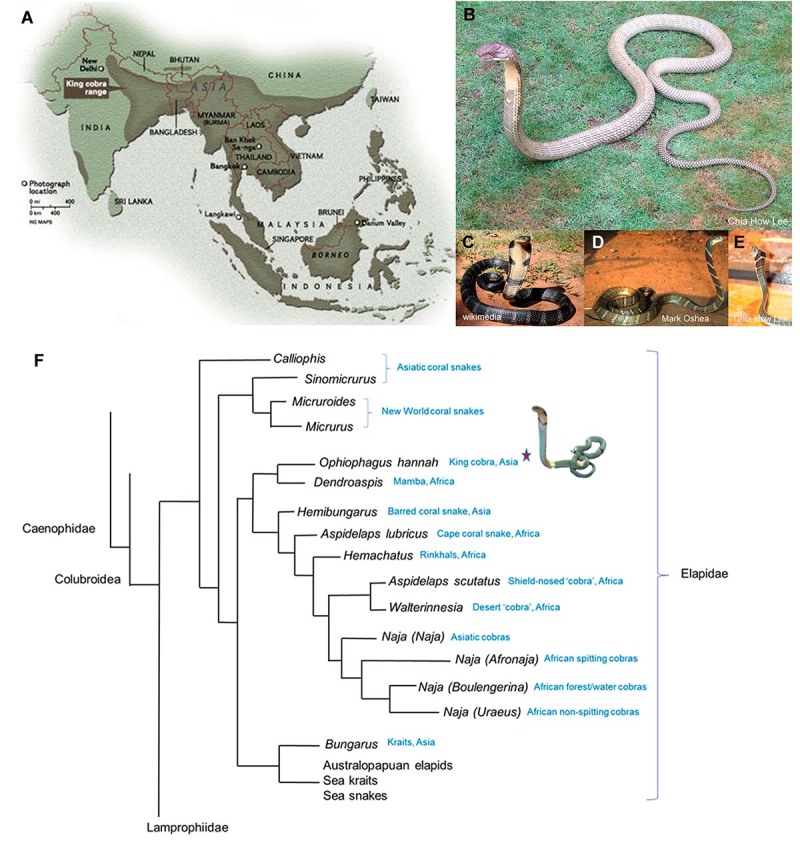
**(A)** Natural distribution of King Cobra in Southeast Asia, southern China, north and north-eastern India, and the Western Ghat of India. **(B)** Adult King Cobra, Malaysia, with pale olive coloration (photo by Chia How Lee, reprinted with permission). **(C)** Adult King Cobra, likely Western Ghats of India, in glossy black color with cross-bars (photo by Michael Allen Smith from Seattle, USA, CC BY-SA 2.0, via Wikimedia Commons). **(D)** Adult King Cobra, Odisha State, eastern India, with brownish bronze coloration and crossbars (photo by Mark O'Shea, https://www.markoshea.info/oba3-1_india01.php, reprinted with permission). **(E)** Juvenile King Cobra (Malaysia) in growth transition, with crossbars which will disappear into adulthood (photo by Chia How Lee, reprinted with permission). **(F)** Schematic representation of the phylogenetics of King Cobra in relation to extant elapids (tree simplified from Figueroa et al. [19]).

King Cobra is the world’s largest venomous snake, with adults capable of growing up to 6 meters in length. It widely distributes throughout Southeast Asia on the Malay Archipelago (to the exceptions of territories located eastward from the Indonesian islands of Java and Sulawesi), in southern China (including the insular Hainan Province and Hong Kong SAR), and in parts of India (in the northern and eastern parts of India, and disjunctly, in southwestern India along the Western Ghats of Tamil Nadu) [15] (Figure 1A). Depending on the geographical origin, habitat and stage of growth, King Cobras can exist in an array of different colors, including glossy black, pale olive, gray, brownish-green or yellowish golden, with or without white crossbars or chevrons on the body (Figure 1B-1E). King Cobras usually inhabit tropical forests although specimens were also sighted in a wide range of habitats, including mangrove forest, highlands, wetlands, scrublands and anthropogenically modified environments such as agricultural fields [15-17]. 

The taxonomy of King Cobra was first described as *Hamadryas hannah* (Cantor) in 1836. The genus *Ophiophagus* was proposed by Günther in 1864, aptly attributing the name to its propensity to eat snakes (*Ophio*-: snake, -*phagous*: eating, according to the Greek etymology). *Ophiophagus hannah* was formally accepted as the valid name for King Cobra in 1945 [18]. Although having a “cobra” in its common name and displaying hooding feature, the King Cobra is not a “true cobra” as it differs on several anatomical characters from those of the *Naja* genus; instead, it was more closely related to mambas (*Dendroaspis* spp.), based on more recent genetic analysis of its phylogeny [19] (Figure 1F). It is currently the monotypic species of the *Ophiophagus* genus, although King Cobras from various regions may be part of a larger species complex. For instance, mitochondrial DNA analysis on King Cobras in Thailand suggested a deep phylogenetic divergence between the northern and southern specimens [20]. In fact, besides variation in morphology and coloration within the species of King Cobra, the venom properties in terms of composition and biological activities tend to vary greatly across specimens from different geographic locations [21,22]. However, polymorphism and venom variation are also known to occur without phylogenetic implication, and serve no indication of speciation [23]. Hence, further phylogenomic study based on more specimens from different geographical populations of King Cobra is needed to shed light on its true taxonomy.

**Figure 2.**
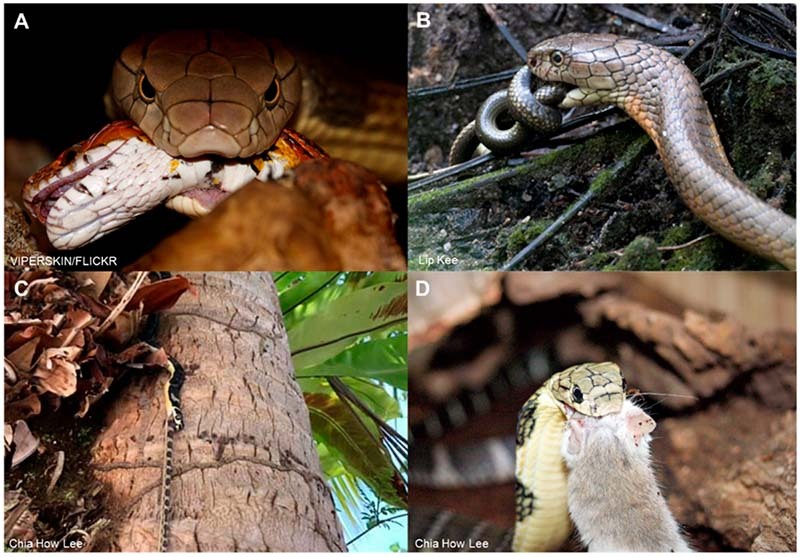
Feeding behavior of King Cobra. Ophiophagy is observed in **(A, B)** adult and **(C)** juvenile King Cobras, feeding on a corn snake, Gerald’s water snake, and bridle snake, respectively (A, photo by viperskin, CC BY-NC-SA 2.0, via Flickr; B, photo by Lip Kee, CC BY-SA 2.0, via Flickr; C, photo by Chia How Lee, reprinted with permission). These were smooth-scaled preys that King Cobras prefer over snakes with keeled scales. **(D)** Opportunistic feeding on rodents, in captive environments (photo by Chia How Lee, reprinted with permission).

The King Cobra primarily feeds on other snakes, both venomous and non-venomous, but it probably also feeds on other reptiles and small mammals when food is scarce (Figure 2). A broad range of preys has been documented including rat snakes, pythons, cobras, kraits, vipers and other King Cobras - A cannibalistic trait is present in this species. Though a snake-eating species, King Cobra is not fully immune to snake venoms, as it can be envenomed and killed by another King Cobra or venomous snakes. Almost exclusively diurnal, the King Cobra is an active predator capable of traveling a long distance, tracking its prey over a prolonged duration [24]. This day-traveler also contributes to the low incidence of envenomation in humans as accidental encounter is less frequent compared with the *Naja* species (cobras) which are relatively more aggressive, and ambushing vipers/pit vipers which are resting motionless and often camouflaged. Additionally, despite its fearless and aggressive reputation, field scientists often described the King Cobra as a placid snake, only manifesting aggressive behavior when disturbed or cornered, while preferring visual and acoustic threats display over attack. Typically, as a sign of warning, it would raise its upper body above the ground, spreading its hood while emitting a loud growling hiss [24,25].

## Sociocultural impact and interaction with humans

**Figure 3.**
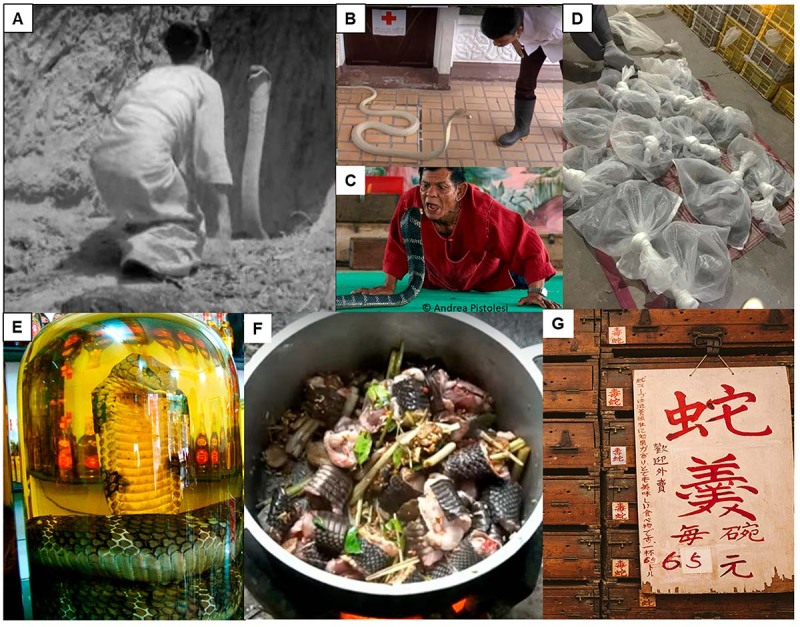
Relationship between King Cobra and humans. **(A)** Footage from ‘*Wheels across India*’ (Armand Denis, Leila Roosevelt; 1940) of a religious ceremony held in Burma (Myanmar) involving a priestess attempting to kiss a King Cobra on its head three times to obtain the favor of the Snake God of Fertility. **(B)** Snake educational show at the snake farm of Queen Saovabha Memorial Institute, Bangkok (screenshot from a YouTube clip: www.youtube.com/watch?v=fWh-XOKJgxM). **(C)** Snake show as a touristic attraction: performer at the Ban Khok Sa-nga King Cobra Village (Thailand) attempting to hold the King Cobra’s head in his mouth (photo by Andrea Pistolesi, https://andreapistolesi.wordpress.com/2010/02/23/who-is-the-snake-who-is-the-victim/). **(D)** Bags of King Cobras in a wildlife trade market, China. **(E)** King Cobra medicinal liquor (photo by Viethavvh, CC BY-SA 3.0, via Wikimedia Commons). **(F)** Village cooking of King Cobra, a source of animal protein in rural areas (screenshot from a YouTube clip: www.youtube.com/watch?v=2Ut0KJv16hg). **(G)** Menu with “snake thick soup” placed against century-old wooden drawers marked with “venomous snake” (where live snakes were kept) in a restaurant, Hong Kong (©zolimacitymag.com, all rights reserved). Such exotic foods are considered a delicacy and traditional health tonic.

The King Cobra holds cultural importance in many Asian populations. It is depicted in the Hindu mythology along with cobras (*Naja* genus), and are considered as the descendant of Nagas, serpent/snake deities. In the 1940 documentary film ‘Wheels across India’, Armand Denis recorded a religious ceremony in Burma (Myanmar), where a priestess was kissing a King Cobra on its head several times and making an offering to the snake as the God of Fertility (Figure 3A). Snake performances are still held in many parts of Asia to this day for both educational and touristic purposes, such as that conducted in the Snake Farm of the Queen Saovabha Memorial Institute, Bangkok (Figure 3B). In Ban Khok Sa-nga (Thailand), also known as the King Cobra Village, residents commonly own King Cobra and snake performances are held daily (Figure 3C). In parts of Asia, snakes including King Cobra are hunted, farmed and traded for food, leather and medicinal uses which are believed to offer health and lifestyle benefits, from fatigue relief to boosting virility (Figure 3D-3G). 

The King Cobra is currently listed in CITES Appendix II, and globally “Vulnerable” by the International Union for Conservation of Nature IUCN, notwithstanding considerable variation of local assessment in different countries. Further taxonomic revision will likely re-define King Cobra into a species complex, and inevitably aggravate the status of each newly erected species.

**Figure 4.**
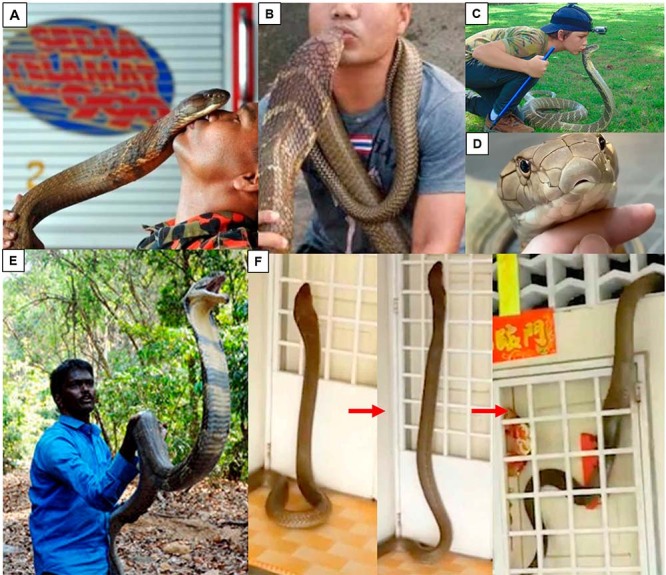
**(A-D)** Posting on social media showing close contact with the King Cobra. Sources: A-B, “whispering” (from www.facebook.com/wira.penyelamat.3); C, kissing (screenshot from a YouTube clip, www.youtube.com/watch?v=qN5x9raeaMI); D, petting and stroking (screenshot from a YouTube clip, www.youtube.com/watch?v=sX6DS98BhUk). **(E)** A snake catcher demonstrating the classical pose of “holding up the King Cobra” barehandedly (screenshot from a YouTube clip https://www.youtube.com/watch?v=0ypL-l2laPQ). **(F)** Snake intrusion alert on a hot day: a King Cobra was spotted slithering into a house in Malaysia (screenshot from a YouTube clip: www.youtube.com/watch?v=F6L_YaWxIA4).

King Cobra is also a favorite subject of many snake enthusiasts, and “internet celebrities” for posting their interaction with the deadly snake on social media. This amateur practice is gaining popularity in recent years but it is undoubtedly dangerous, as the interaction typically involves bare-hand handling of the snake and exaggerated actions such as petting and kissing the snake (Figure 4). Unnecessary handling and accidental provocation of the snake would lead to its defensive bite and envenomation. Although these people could be well-trained snake handlers, the practice of unnecessary posing (barehand handling, petting, kissing) with the venomous snakes in social media is controversial, as non-trained persons might be tempted to imitate the action. Climate change (global warming, flash floods) and other anthropogenic factors that result in the clearing of wildlife habitat also contribute to the increasing incidence of snake intrusion into human settlement, resulting in human-snake conflicts. These will emerge as the leading causes of King Cobra envenomation in the future, considering that awareness practice and protective equipment may reduce the risk of snakebite among the rural and agricultural populations.

## Medical importance

King Cobra is classified as a venomous snake of secondary medical importance (WHO Category 2). By definition, venomous snakes listed in Category 2 are highly venomous species capable of causing morbidity, disability or death, for which exact epidemiological or clinical data may be lacking; and/or are less frequently implicated (due to their activity cycles, behavior, habitat preferences or occurrence in areas remote to large human populations) [1]. The majority of envenomation cases resulted from mishandling, and professions working in close contact with venomous snakes (*e.g.*, venom extraction worker, reptile caretaker, snake rescuer) are thus exposed to a higher risk of envenomation [26,27]. Clinical cases of King Cobra envenomation reported in scientific literature consisted of bites occurring in snake handling environment [26-31]. The notorious popularity of this species and its consequent usage in recreational performances (*e.g.*, snake charming, snake dance) also led to documented envenomation cases [31]. News media also commonly reported casualties arising from such close contacts [32-34].

## Clinical cases of King Cobra envenomation

The global epidemiological data of snakebite is scarce. For envenomation caused by King Cobra, there were only nine clinical reports available in the literature published between 1971 and 2020. Of these, three cases were reported from Myanmar, one each from Thailand and Malaysia, and four occurred outside Asia: two in the USA, one in the UK and one in the Netherlands. King Cobra envenomation in humans typically manifests through local tissue damage and systemic neurotoxicity, with a constellation of signs and symptoms similar to that caused by cobras (*Naja* spp.) in Asia. Envenomed patients commonly develop intense local reactions characterized by pain and swelling resulting in tissue necrosis, and descending paralysis that leads to respiratory failure - the principal mode of fatality in envenomation by King Cobra [35]. 

Table 1 summarizes the reported cases of King Cobra envenomation in humans, with details of the clinical manifestation and the use of antivenom (if any) for treatment. All cases exhibited local envenomation effects at varying degrees of severity, most were presented with noticeable signs of aggravating inflammation (pain and intensive swelling around the bite site, which could be extending to the entire bitten limb and adjacent body structures) [31,36]. In severe envenomation, tissue necrosis developed requiring surgical interventions and amputation [37]. Non-specific systemic effects were also reported (*e.g.*, dizziness, vertigo, nausea), accompanied with signs of neuromuscular paralysis such as ptosis, limb weakness, dysphagia, dysarthria and flaccid paralysis. Paralysis of respiratory muscle results in asphyxia and generalized hypoxia, with death ensues from multiorgan failure [26,27,31,36,38]. 

**Table 1.** Clinical cases reported on *Ophiophagus hannah* envenomation and treatment.

Country	Cases	Local effect	Systemic effect	Intervention	Reference
Myanmar	3	Pain, extensive swelling, necrosis	Dizziness, ptosis, ophthalmoplegia, respiratory failure, flaccid paralysis	Artificial respiration; antivenom: OhMAV (20 vials), surgical debridement	Tin et al. [31]
UK	1	Swelling, ischemia	Dizziness, ptosis, dysphagia, hypertension, bradycardia	Artificial respiration; antivenom: OhMAV (20 vials)	Veto et al. [26]
USA	1	Pain, swelling	Visual hallucination, lethargy, headache, myalgias, periodic syncope	Antivenom: OhMAV (50 vials)	Wetzel and Christy [27]
Malaysia	1	Pain, extensive swelling	Dizziness, nausea, vomiting, blurred vision, dysphagia, dysarthria, flaccid paralysis, tachycardia	Artificial respiration; antivenom: OhMAV (33 vials)	Ismail et al. [36]
Thailand	1	Pain, extensive swelling, infection	Drowsiness, ptosis, dysphagia, dysarthria, syncope, hypotension, respiratory failure	Artificial respiration; antivenom: OhMAV (115 vials)	Ganthavorn [28]
USA	1	Pain, swelling	Confusion, ptosis, dysphagia, dysarthria, flaccid paralysis	Artificial respiration; antivenom: OhMAV (15 vials)	Gold and Plye [29]
The Netherlands	1	Blistering, cellulitis, digital ischemia and skin necrosis	None	Wound debridement, digital amputation	Imran et al. [37]

OhMAV: Thai *Ophiophagus hannah* Monovalent Antivenom.

In all cases reported, the diagnosis was made clinically through history taking or identification of the biting snake. The treatment consisted of local effect management through specialized wound care and control of secondary infection, and treatment of systemic neurotoxicity with intubation and assisted ventilation [26,28,29,31,36]. The antivenom specific to King Cobra, *i.e., Ophiophagus hannah* Monovalent Antivenom (OhMAV) produced in Thailand was administered in eight out of the nine cases, indicated by the onset of neurological manifestation. The antivenom treatment was somewhat effective in reversing the systemic toxicity at high dosage ranging from 20 to 115 vials, indicating limited neutralization potency of the antivenom.

## Management and potential challenges

**Figure 5.**
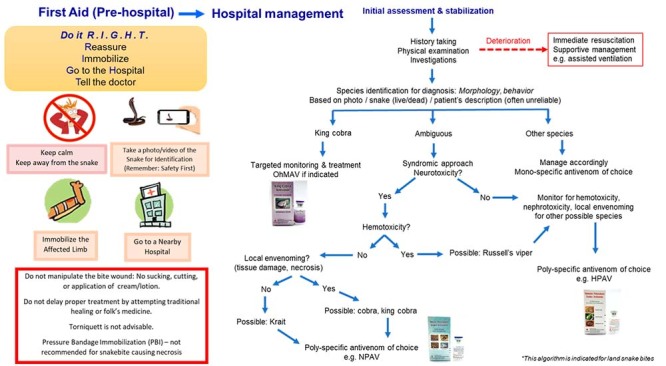
First aid of snakebite and a simplified algorithm for clinical management of snakebite (with a focus on King Cobra) envenomation. OhMAV: *Ophiophagus hannah* Monovalent Antivenom; NPAV: Neuro Polyvalent Antivenom; HPAV: Hemato Polyvalent Antivneom.

In general, when a bite occurs, the first thing the injured should do is to move beyond the striking distance of the snake (to prevent repeated bite). The management begins with first aid as part of pre-hospital care, while the patient is transferred to the nearest medical facility that manages snakebite as soon as possible. Figure 5 illustrates the basic first aid of snakebite the public should be aware of, and a simplified algorithm for clinical management of snakebite envenomation (with a focus on King Cobra bite). Principles and issues pertaining to the management are described below.

### Pre-hospital first aid

The use of a tight (arterial) tourniquet, as widely practiced by many folks using a belt, shoelace, clothing or even fence wire, severs the blood and lymphatic circulation distally and thus potentially prevent or reduce the systemic spread of venom from the bitten site. This practice, however, is highly controversial and now discouraged especially in bites that can cause tissue damage, as tourniquets can concentrate the venom at the bitten site, promoting severe necrosis, gangrene, and resulting in the need to amputate, besides the dangers of “sudden release” (bolus of venom being released systemically leading to overwhelming toxic activity when the tourniquet is removed abruptly). Pressure bandage immobilization (PBI), much advocated by Australian practitioners, is an alternative that effectively reduces lymphatic drainage (the main route of venom spread) without compromising blood circulation. However, PBI too has a limited role in cytotoxic and hemotoxic bite, again, for the risk of worsened local tissue damage as seen in bites caused by most venomous Asiatic snakes, including the King Cobra. Thus, light bandaging along with immobilization of the bitten limb has been a more common practice here while the victim is being transferred at the soonest for medical care. 

### Species identification

The identification of species is crucial for the selection of an appropriate antivenom, which is usually species-specific. The identification of snake species is, however, clinically challenging. Diagnosis based on the victim’s description is usually less reliable, while captured specimen of the biting snake may not be always available - in fact, it is advisable not to capture the snake to avoid secondary bite. Increasingly, people have the possibility of getting a photo or video of the offending snake in a bite by using the cell phone. This should be recommended, followed by the consultation with an expert who is able to identify the snake.

Alternatively, the syndrome-based approach is useful for diagnosis when the description of snake is unclear, or the offending snake photo or specimen is unavailable. This however can be equally challenging in areas where several sympatric species that produce similar envenoming effects. In Asia, envenomation by the majority of cobra and krait species (land snakes) produces similar neuromuscular paralytic effect, as with the King Cobra. The kraits, nonetheless, do not cause a significant local envenoming effect, and thus it can be relatively easy to rule out krait envenoming based on clinical observation. Between cobras (*Naja* spp.) and King Cobra, the envenoming effects are rather similar, yet requiring totally different specific antivenom for effective treatment. From the practical standpoint, King Cobra envenomation typically occurs in captive environments and rarely in the wild, therefore species identification has not been much of a problem in King Cobra envenomation, and the mono-specific King Cobra antivenom can be opted as the first-line treatment accordingly. In a worse scenario where the neurotoxic species cannot be identified, the patient will have to rely on the availability of a polyvalent antivenom that is able to cover the different neurotoxic species in the region (Neuro Polyvalent Antivenom, product of Queen Saovabha Memorial Institute, Bangkok). A brief guide on the syndromic identification of King Cobra bite and antivenom recommendation is illustrated in Figure 5. 

### Antivenom production and efficacy

The Thai Red Cross Society, Queen Saovabha Memorial Institute (Bangkok, Thailand) is the only antivenom producer in the world that manufactures the specific antivenom, *Ophiophagus hannah* Monovalent Antivenom (OhMAV), raised against the King Cobra venom of Thai origin. A polyvalent antivenom, Neuro Polyvalent Antivenom (NPAV) that can be used to treat the envenomation caused by four medically important elapids in the region (*Ophiophagus hannah*, *Naja kaouthia*, *Bungarus candidus*, *Bungarus fasciatus*) is also available. These products are commonly used in the region, including Malaysia, as both countries shared similar medically important venomous herpetofauna [39]. In Myanmar, the Philippines, Indonesia and China, local production of antivenom is available to support domestic demand, but none of the antivenoms from these countries covers King Cobra. Anecdotal records showed that non-specific heterologous antivenoms such as antivenoms for cobra (*Naja*) envenomation were used as an alternative to treat King Cobra envenomation in China and Indonesia, but the treatment outcome has been poor. Although hetero-specific antivenoms may cross-neutralize the venoms of closely related species which share compositional and antigenic similarities [40], King Cobra and *Naja* cobras have variable venom antigenicity that limits immunorecognition of toxins and cross-neutralization by antivenom immunoglobulins [22]. Moreover, the use of high doses of less effective hetero-specific antivenom could pose a greater risk of hypersensitivity reaction with fatal outcome to the patients [41].

Snake venoms are integrated phenotypes used for predatory, digestive and defensive purposes, and are subject to evolution with adaptation to changes in ecological niches. Intraspecific variation of snake venom composition is a widely recognized phenomenon, and this has a huge impact on the effectiveness of antivenom used. Compositional variation of snake venom within a species from different locales is often accompanied with variable expression and antigenicity of the key toxins, rendering the antivenom from a single production source less potent or even ineffective [42-45]. Although in some instances, the reversal of toxicity can be achieved by increasing the dosage of antivenom administered, but this inevitably exposes the patient to a greater risk of hypersensitivity reaction, increases the treatment cost and rapidly exhausts the antivenom reserves. In King Cobra envenomation, the effectiveness of OhMAV in regions outside Thailand is questionable when considering the extensive biogeographical distribution of King Cobra. The dosage of antivenom for King Cobra envenomation tends to be exceptionally high (beyond 20 vials), probably because of the need to neutralize a massive amount of venom injected - In author’s experience, King Cobra is capable of delivering more than 1 gram (dry weight) of venom in one bite, and it has the propensity to hold its bite on the bitten subject for a while (several minutes), instead of the bite-and-release strike as seen in other snakes. 

### Treatment of local envenomation

King Cobra envenomation can result in extensive tissue necrosis, which requires serial wound debridement, followed by reconstructive surgery using skin grafting and/or a flap, or, amputation in severe cases [37]. To prevent later sequelae, local tissue damages caused by King Cobra bite are best attended soon with surgical debridement to remove as much venom as possible. However, there is no consensus on the time to conduct such intervention to achieve the most optimal outcome. Early antivenom administration (intravenous) may potentially reduce the spread of venom’s local activity, but the efficacy has not been well established. In cases presenting with severe and progressive swelling, inadequately informed clinicians might view it as compartment syndrome needing fasciotomies, and often the decision to send the patient to surgery for a fasciotomy is taken without the supporting tests to measure the intra-compartmental pressure. In King Cobra envenomation, such radical surgery is unjustified as the swelling is typically subdermal instead of deep tissue as produced by crush injuries. 

## King Cobra venom: composition and bioactivity studies

**Figure 6.**
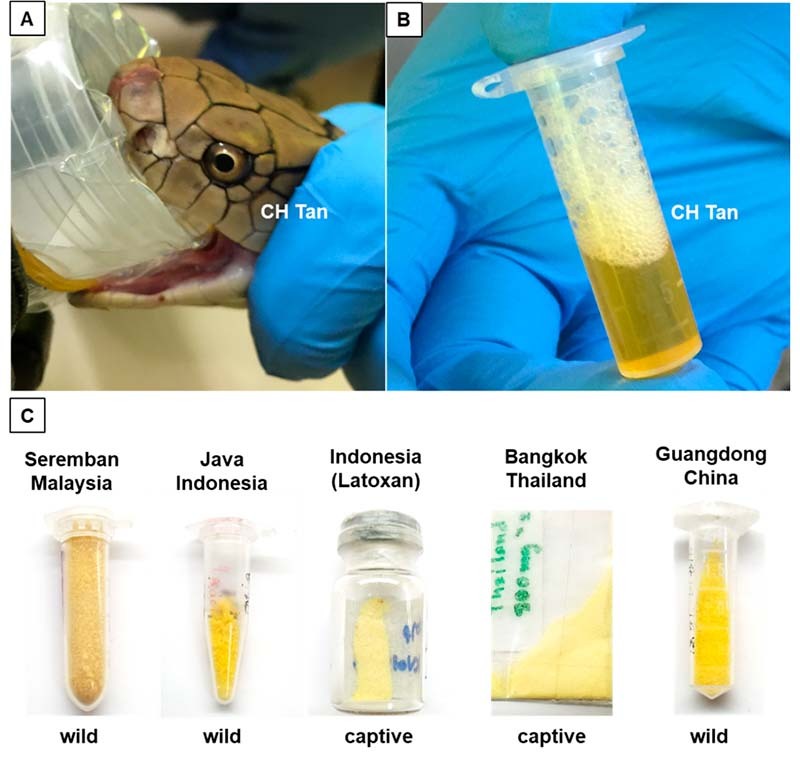
Collection of King Cobra venom. **(A)** Milking of venom by inducing the snake to bite through a film-covered clean container. **(B)** Freshly milked venom with its bright golden color. **(C)** Venom powder in different shades of yellow obtained through lyophilization for long-term storage.

The studies are usually conducted on reconstituted venom powder, obtained through lyophilization (freeze-drying) or desiccation of crude venom milked from live snakes. King Cobra venom can be readily collected by inducing the snake to bite through a film covering a clean container, or, by directly placing its fangs over the edge of a collecting device. The venom of King Cobra is typically golden yellow in color, often viscous, and lyophilization yields powder with comparable coloration in different shades of yellow which is indicative of the presence of a flavin adenine dinucleotide (FAD)-containing flavoenzyme, L-amino acid oxidase (LAAO) (Figure 6). 

The investigation of King Cobra venom properties dated back to the 1960s when Tu et al. [46] studied the hemorrhagic and proteolytic activities of the venom. Various bioactive components had been isolated from the venom and characterized, including various proteins from the families of three-finger toxins (3FTx) [47,48], snake venom metalloproteinases (SVMP) [49], cysteine-rich secretory proteins (CRiSP) [50], phospholipases A_2_ (PLA_2_s) [51], L-amino acid oxidases (LAAOs) [52], Kunitz-type serine protease inhibitors (KSPIs) [53], cobra venom factors (CVFs) [54] and ohanin (vespryn) [55]. The global protein profile of King Cobra venom was only available with the advent of proteomics, which attempted to unveil the variety and abundances of all toxins (proteins) in the venom comprehensively. Thus far, a total of nine proteomic studies on King Cobra venom have been reported, with noticeable discrepancies in details. The proteomes were summarized in Table 2 and Table 3 for profiling based on quantitative and non-quantitative approaches, respectively.

**Table 2.** Quantitative proteomic profiling of King Cobra (*Ophiophagus hannah*) venom.

Reference	Petras et al. [56]	Tan et al. [57]	Liu et al. [58]
Geographical origin	Indonesia, unspecified locale	Malaysia, Seremban (southwestern peninsula)	Indonesia, unspecified locale
Specimen habitat and source	Captivity, commercial source (Latoxan)	Wild	Captivity, commercial source (Latoxan)
Number of specimens	Unspecified	1	A few
Specimen growth state	Unspecified	Adult	Adult
Specimen sex	Unspecified	Unspecified	Unspecified
Methodology	C_18_ reverse-phase HPLC Reducing SDS-PAGE In-gel digestion LC-MS/MS	Reducing SDS-PAGE In-gel digestion LC-MS/MS	C_18_ reverse-phase HPLC Reducing SDS-PAGE In-gel digestion LC-MS/MS
**Quantitation**	**Relative protein abundance of toxin (% of total venom proteins) and number of protein subtype (number in parentheses)**		
**Toxin family**			
3FTx	64.2 (≥ 20)	43.0 (13)	60.65 (21)
*Long neurotoxin*	*37.1*	*26.7*	*~33.5*
*Short neurotoxin*		*10.2*	*~11.9*
*Weak toxin/neurotoxin*	*13.7*	*3.9*	*~3.4*
*Muscarinic toxin-like protein*	*4.7*	-	-
*Cytotoxin/cardiotoxin*	*8.7*	*0.5*	*11.9*
SVMP	11.9 (2-3)	24.4 (12)	15.63 (11)
PLA_2_	2.8 (5)	4.0 (1)	3.66 (3)
CRiSP	6.5 (1)	8.7 (1)	7.46 (2)
KSPI	3.3 (1)	1.0 (1)	2.93 (1)
LAAO	0.5 (1)	5.7 (2)	-
CVF / OVF	-	2.8 (4)	-
Vespryn	1.1 (1)	5.7 (1)	1.83 (2)
ILGF	0.7 (1)	-	-
NP	0.2 (3)	-	-
SVSP	-	0.7 (1)	-
Cystatin	Trace (1)	0.7 (1)	-
PDE	-	1.1 (2)	-
NGF	-	0.8 (1)	-
AChE	-	0.1 (1)	-
Neprilysin	-	0.7 (2)	-
PLB	-	0.2 (1)	-
5NT	-	0.4 (1)	-
VEGF	-	-	-
CTL	-	-	-
Waprin	-	-	-
Others	-	-	7.84 (9) Unknown protein

3FTx: three-finger toxin; 5NT: 5' nucleotidase; AChE: acetylcholinesterase; CRiSP: cysteine-rich secretory protein; CTL: C-type lectin; CVF: cobra venom factor; HPLC: high performance liquid chromatography; ILGF: insulin-like growth factor; KSPI: Kunitz-type serine protease inhibitor; LAAO: L-amino acid oxidase; LC-MS/MS: liquid chromatography-tandem mass spectrometry; NGF: nerve growth factor; NP: natriuretic peptide; OVF: *Ophiophagus* venom factor; PAGE: polyacylamide gel electrophoresis; PDE: phosphodiesterase; PLA_2_: phospholipase A_2_; PLB: phospholipase-B; SVMP: snake venom metalloproteinase; SVSP: snake venom serine protease; VEGF: vascular endothelial growth factor.Note: 3FTx comprises of long neurotoxin, short neurotoxin, weak toxin/neurotoxin, muscarinic toxin-like protein and cytotoxin/cardiotoxin.

**Table 3:** Non-quantitative proteomic profiling of King Cobra (*Ophiophagus hannah*) venom.

Reference	Vonk et al. [59]	Vejayan et al. [60]	Danpaiboon et al. [61]	Melani et al. [62]	Kunalan et al. [63]	Wongtay et al. [64]
Geographical origin	Indonesia, Bali Island	Malaysia, specific locale unknown	Thailand, specific locale unknown	Malaysia, specific locale unknown	Malaysia, Perlis (northwest Malaysia)	Thailand, Phetchabun, Songkhla, Suratthani
Specimen habitat and source	Unspecified	Unspecified, from local vendor	Captivity, from Queen Saovabha Memorial Institute	Captivity, from Kentucky Reptile Zoo in USA	Captivity, from a snake enthusiast	Unclear, captive-born was included
Number of specimens	1	Unspecified	Unspecified	2	Unspecified	One per locale, total of 3
Specimen growth state	Adult	Unspecified	Unspecified	Unspecified	Unspecified	Unspecified
Specimen sex	Unspecified	Unspecified	Unspecified	Unspecified	Unspecified	Unspecified
Methodology	Whole venom in-solution digestion LC-MS/MS	2D-PAGE In-gel digestion MALDI-TOF MS	2D-PAGE In-gel digestion LC-MS/MS	GELFrEE Solution isoelectric focusing In-solution digestion LC-MS/MS	Gel-filtration chromatography In-solution digestion LC-MS/MS	2D-PAGE In-gel digestion MALDI-TOF MS
**Detection**	**Occurrence of toxin in percentage^1^ (number of proteins in parentheses)**	**Number of proteins**	**Number of proteins**	**Number of proteins (number of distinct proteoforms in parentheses)**	**Occurrence of toxin in percentage^1^ (number of proteins in parentheses)**	**+: Detected**
**Toxin family**						
3FTx	41.1% (30)	7	16	57 (29)	31.0% (55)	+
SVMP	24.7% (18)	-	4	14	25.0% (44)	-
PLA_2_	5.5% (4)	4	5	10 (9)	5.0% (9)	+
CRiSP	6.8% (5)	-	3	3 (3)	9.0% (15)	+
KSPI	4.1% (3)	-	3	2 (8)	3.0% (6)	-
LAAO	1.4% (1)	-	1	2	2.0% (3)	-
CVF / OVF	5.5% (4)	1	1	4	4.0% (7)	-
Vespryn	1.4% (1)	1	2	3 (4)	1.0% (2)	+
ILGF	-	-	1	3	3.0% (5)	-
NP	1.4% (1)	-	-	-	-	+
SVSP	4.1% (3)	1	-	1	1.0% (2)	-
Cystatin	-	-	-	-	-	-
PDE	1.4% (1)	-	1	-	6.0% (11)	-
NGF	-	-	-	-	1.0% (2)	-
AChE	-	-	-	-	1.0% (2)	-
Neprilysin	-	-	-	-	1.0% (1)	-
PLB	-	-	-	-	1.0% (1)	-
5NT	-	-	1	-	2.0% (3)	-
VEGF	2.7% (2)	-	-	3	1.0% (1)	+
CTL	-	-	-	-	1.0% (2)	+
Waprin	-	-	-	1	-	-
Others	-	1 Thioredoxin	4 Hypothetical 4 Other	2 Disintegrin 25 non-toxin 1 SVGF	2.0% (3) SVGF 1.0% (2) Endonuclease	+ (unspecified)

3FTx: three-finger toxin; 5NT: 5' nucleotidase; AChE: acetylcholinesterase; CRiSP: cysteine-rich secretory protein; CTL: C-type lectin; CVF: cobra venom factor; GELFrEE: Gel-eluted liquid fraction entrapment electrophoresis; ILGF: insulin-like growth factor; KSPI: Kunitz-type serine protease inhibitor; LAAO: L-amino acid oxidase; LC-MS/MS: liquid chromatography-tandem mass spectrometry; NGF: nerve growth factor; NP: natriuretic peptide; OVF: *Ophiophagus* venom factor; PAGE: polyacylamide gel electrophoresis; PDE: phosphodiesterase; PLA_2_: phospholipase A_2_; PLB: phospholipase-B; SVGF: snake venom growth factor; SVMP: snake venom metalloproteinase; SVSP: snake venom serine protease; VEGF: vascular endothelial growth factor.

+: indicates detection; -: indicates not reported.

^1^ A ratio obtained whereby the number of proteins in a family is divided by the total number of all proteins detected in proteome.

### Three-finger toxins (3FTx)

Molecular cloning and recent -omic studies demonstrated the diversity of three-finger toxins that include alpha-neurotoxins, muscarinic toxin-like proteins (MTLPs), weak neurotoxins (WNTXs) and cardiotoxins/cytotoxins (CTXs) in King Cobra venom [47,56,57,59]. Of these, the alpha-neurotoxins (long neurotoxin, LNTX and short neurotoxin, SNTX) are the most diversely and abundantly expressed (Table 2 and Table 3). SNTX and LNTX are the main neurotoxins with high lethal activity (median lethal dose, LD_50_ ~0.05-0.20 µg/g, in mice), commonly found in the venoms of most Asian elapids (*Naja* cobras, kraits, sea snakes) [57,65-67]. MTLP and WNTX belong to the short-chain and non-conventional subfamilies of 3FTx, respectively. MTPLs interact with muscarinic acetylcholine receptors (mAChR) [68] while WNTXs bind to both muscular and neuronal nAChR but lack lethal activity (LD_50_ = 4-80 µg/g) [69,70]. CTXs, on the other hand, exhibit a more diverse array of pharmacological activities. CTX isolated from *Naja* cobra venoms typically show cytolytic and cytotoxic activities [71]. The CTX from King Cobra venom, *i.e.*, beta-cardiotoxin (β-CTX), was shown to bind to β-1 and β-2 adrenergic receptors of cardiomyocytes, and exhibit negative chronotropic activity [48,72]. Cardiac complication has not been well established in clinical envenomation caused by King Cobra, presumably due to the low abundance of this toxin in the venom. 

Quantitative proteomics showed that 3FTxs are the most abundant toxins in King Cobra venom from Malaysia (accounting for 43% of the total venom protein; 13 subtypes) [57] and Indonesia (64.2%, ≥ 20 subtypes [56], and 60.65%, 21 subtypes [58]). Among these, α-NTXs accounted for the majority of 3FTx (Table 2). Consistently, LNTX were present at a higher relative abundance than SNTX in the Malaysian (26.7% LNTX, 7.5% SNTX) and the Indonesian (33.5% LNTX, 11.9% SNTX) specimens. The abundance of CTX was rather variable, constituting 8.7-11.9% of total venom proteins in the Indonesian King Cobra venom [56,58] while only found at a negligible amount in the Malaysian King Cobra venom proteome (0.5%) [57]. Interestingly, β-CTX was recently isolated from the Thai King Cobra venom, also at a very low recovery rate (0.53% w/w). The existing data tend to indicate that the King Cobras from Malaysia and Thailand, and perhaps the Indochinese Peninsula, probably share a venom phenotype in which very low amount of CTX is expressed, in contrast to the insular populations from Indonesia. The abundances of non-conventional 3FTx, on the other hand, were consistent in the Malaysian and Indonesian King Cobra venom proteomes (~4%). In proteomic studies where relative abundances of proteins were not reported [59-64], 3FTxs overall showed the highest number of toxin subtypes detected in the venom (Table 3).

### Snake venom metalloproteinases (SVMPs)

The SVMPs are multi-domain zinc-dependent enzymes of varying molecular weights. They are the principal toxins responsible for vasculature damage and may interfere with hemostasis, contributing to hemorrhage and coagulopathy in envenomation caused by vipers and pit vipers [73,74]. Compared to most cobra venoms in which SVMPs were little (< 1% of total venom proteins), SVMPs in King Cobra venom were found to be more diverse and abundantly expressed, accounting for 24.4% (12 subtypes) [57], 11.9% (2-3 subtypes) [56] and 15.63% (11 subtypes) [58] of total venom proteins in three separate Southeast Asian samples (Table 2). All SVMPs identified in King Cobra venom belong to the PIII class, which is made up of a metalloproteinase (M) domain, a disintegrin (D) domain and a cysteine-rich (C) domain. PIII-SVMPs are generally more potent hemorrhagin than PI-SVMPs (containing only M domain) and PII-SVMPs (containing M and D domain) [73]. The genomic and transcriptomic studies of King Cobra [57,59] revealed that the metalloproteinase of their PIII-SVMPs have only six cysteine residues, in contrast to most viperid SVMPs whose M domains contain an additional seventh cysteine residue at varying positions. Previous studies correlated the presence of the seventh cysteinyl residue in some SVMPs to vascular apoptosis-inducing activity, and indicated that in PIIIa subclass, the seventh cysteinyl residue at position 195 may be implicated in alternative disulfide bond pairing and proteolytic processing whereby proteolysis/autolysis produces a biologically active DC (disintegrin- and cysteine-rich) domain [73]. The evolutionary and medical implication of the sequence variation seen in King Cobra SVMP (PIII) has yet to be dissected out. Clinically, hemorrhagic effect is not observed in human envenomation caused by King Cobra, although its venom has been found to show hemorrhagic activity in rabbits and hares [75]. In addition, a 63−66 kDa hemorrhagin (with SVMP biochemical properties) was isolated from King Cobra venom previously and shown to exhibit species-sensitive hemorrhagic and lethal activities in rabbits, while its toxicity in mice was minimum [76]. Apparently, the pathophysiological role of King Cobra SVMP in human envenomation is under-explored, although its substantial abundance suggests involvement in local tissue damage and necrosis resulting from their inflammatory and proteolytic activities which are instrumental for foraging and digestive purposes. 

### Cysteine-rich secretory proteins (CRiSPs)

Cysteine-rich secretory proteins (CRiSPs) were the third most abundant protein family detected in King Cobra venom proteomes, constituting 6.5% to 8.7% of total venom proteins [56-58]. Ophanin, a CRiSP isolated from *O. hannah* venom [50], was commonly identified in the venom proteomes reported. The biological activities of CRiSP include the inhibition of voltage-dependant Ca^2+^ channels and blockade of smooth muscle contraction [50]. Despite its substantial abundance, the pathogenic role of Ophanin and other CRiSP proteoforms in King Cobra envenomation remains unknown. 

### Phospholipases A2 (PLA2s)

In King Cobra venom proteome, PLA_2_s were represented by a rather small number of subtypes and a low abundance (2.8-4% of total venom protein) [56-58]. The PLA_2_s in King Cobra venom consist of two distinct subgroups: group IA, including acidic PLA_2_s which exhibited mild proinflammatory activity but were non-lethal to mice at a high dose of 10 µg/g [77], and group IB with weak lethality and cardiotoxicity in mice [51]. While snake venom PLA_2_s are commonly present in most snake venoms implicated in the pathophysiology of envenomation, the function of PLA_2_ in King Cobra venom is likely ancillary without direct lethal activity. 

### L-amino acid oxidases (LAAOs)

King Cobra venom is a rich source of snake venom LAAO - the intense yellow coloration of the venom reflects the high content of FAD-containing LAAO in the venom (Figure 6). LAAO was reported to constitute close to 6% of total venom proteins in the Malaysian King Cobra venom proteome [57]. In the venom proteome of Indonesian specimen, the enzyme was either undetected [58] or present at only 0.5% of total venom proteins [56], although the Indonesian King Cobra venom too exhibits strong LAAO enzymatic activity [21], and shows yellow coloration (Figure 6). The substantial amount of LAAO in the Malaysian King Cobra venom [57] supported its strong enzymatic activity [21], and suggests the involvement of this enzyme in the development of local tissue damage (cytotoxic activity) and pain in King Cobra envenomation [78]. 

### Minor toxin proteins

Several protein families of low abundances had been reported in proteomic studies of King Cobra venom. Among these were Kunitz-type serine protease inhibitors (KSPIs), which accounted for 1.0-3.3% of total venom proteins [56-58]. Snake venom KSPIs exhibit various biological functions including trypsin/chymotrypsin inhibition, anticoagulant and neurotoxic activity [79-81]. The role of KSPI in King Cobra venom, however, remains not well characterized to date. Vespryn, a novel snake venom protein family, was reported at a relative abundance between 1% and 5% of total venom proteins. Ohanin is a vespryn protein specific to King Cobra and capable of inducing hypolocomotion in mice. The absence of peripheral neurotoxicity of vespryn, nonetheless, implies a direct action on the central nervous system [55]. The hypolocomotion effect of ohanin probably contributes toward the predatory function of King Cobra venom.

Other minor protein families were reported rather variably in the King Cobra venom proteomes. Four subtypes of cobra venom factors (CVFs), including *Ophiophagus* venom factor (OVF) accounted for 2.8% of the Malaysian King Cobra venom proteome [57] while none was reported in the proteomes of Indonesian specimens [56,58]. CVF/OVF are non-lethal proteins structurally similar to complement C3 homolog (CC3H), and presumably involved in the activation of complement system. The release of C3a and increases vascular permeability and thus, facilitates systemic spread of the venom [82]. Other proteins of minor abundance are present at a relatively low or negligible amount in the venom, and these probably play an ancillary role in the predatory or digestive function of the venom. These minor components include proteins from the families of snake venom serine protease (SVSP) [57,59,60,62,63], phosphodiesterase (PDE) [57,59,61,63], acetylcholinesterase (AChE) [57,63], phospholipase-B (PLB) [57,63], C-type lectin (CTL) [63], 5’-nucleotidase (5NT) [57,60,63], natriuretic peptide (NP) [56,59,64], insulin-like growth factor (ILGF) [56,61-63], nerve growth factor (NGF) [57,63], vascular endothelial growth factor (VEGF) [59,62-64], cystatin [56,57], neprilysin [57,63] and waprin [63]. 

## Reflections on venom proteomics

The proteomics of King Cobra venom sourced from different geographical locales has been studied to varying depths. Specimens studied thus far were from Malaysia (peninsula) [21,57,60,62,63], Indonesia (Java Island and Bali Island) [21,56,58,59], Thailand [21,61,64] and China [21], while distant and disjunct populations from India, Borneo and the Philippines have not been investigated. Comparison of the proteomes revealed remarkable variations in terms of the subtypes and abundances of toxin proteins identified (Table 2). These quantitative and qualitative differences probably reflected true intra-species venom variability of King Cobra from allopatric populations, and therefore were regarded as geographical variation in venom composition. However, the “proteomic differences” across the above studies were more likely due to sampling and technical reasons. Firstly, the origins and sources of the venom samples were diverse: the direct geographic provenance, from which the precise locales within each country is often not disclosed [56,58,60-62]. Information on the age, sex and number of snakes contributed to the venom sample was often lacking or incomplete. Additionally, some samples were either obtained from the wild specimen [57,59,64], or, presumably captive or farmed specimens through commercial serpentaria [56,58,62], or unreported [60,61,63]. On the other hand, the “functional proteomic approach” reported by Chang et al. [21] focused on characterizing enzymatic activities and identification of only 2 protein families (neurotoxins and phospholipases A_2_) without a comprehensive profile of all venom proteins. Possible interference from factors relating to the sex, developmental (ontogenic) stage and habitat of the snake cannot be excluded when addressing the variability of snake venom composition. Comparison of the findings and interpretation for intra-species variation is, therefore, challenging. 

The second and perhaps more influential factor is the methodology of analysis. The different approaches employed in each study resulted in varying depths of proteomic profiling, which could be confounding when comparison is made, resulting in misinterpretation of geographical venom variation. Proteomics in snake venom research aims to comprehensively profile the diversity (how many?) and protein abundance (how much?) of all toxins present in a venom. The identification of high protein diversity is usually achieved using protein decomplexation strategies that deploy chromatographic or electrophoretic separation methods of venom proteins, prior to mass spectrometry analysis [83-86]. The sensitivity of the detection tool and the depth of database used in the bioinformatic analysis play crucial role in this regard. On the other hand, the quantitation methods of protein abundance in venom proteomics varied greatly. Of the nine proteomic studies of King Cobra venom to date, four listed and numbered the proteins identified [60-62,64], while five, in addition to protein name listing, also reported the “percentage” of individual protein or protein family found in the proteome [56-59,63] (Tables 2 and 3). Among the latter five studies that reported protein percentages, three truly quantified the relative protein abundance as percentage in terms of total venom proteins, estimated based on parameters such as peak area under the curve (chromatography), intensity of gel band on electrophoresis, and/or spectral input of peptide ions in mass spectrometry [56-58]. In two other studies including the proteome reported alongside the King Cobra’s genomics [59,63] (Table 3), the “protein percentages” reported indicated the ratio of a protein number, mathematically derived through dividing the number of proteins (those of the same family) by the total number of all proteins detected. Using this simple ratio calculating method, the more diverse a protein family is, the more “abundant” it will appear to be, but this association is not necessarily true and is not compatible with the analysis for “relative protein abundance”. Therefore, the inadequate information of the geographical sample of venom, and the different methods of analysis applied impede meaningful comparison of King Cobra venom proteomes reported in different studies. 

## Variation in King Cobra venom toxicity and neutralization

Earlier, Tan et al. [87] reported no remarkable variation in the toxicity of King Cobra venoms sampled from limited sources (two specimens from peninsular Malaysia, one specimen from southern Thailand, and one from a commercial source of unspecified locality). Chang et al. [21] subsequently compared the lethality and enzymatic activities of King Cobra venoms from five geographic locales, *i.e.*, Thailand, Malaysia, Indonesia and two Chinese localities (Hainan and Guangxi) (the number of snakes in each geographical sample was not known). Their results demonstrated marked variations in the enzymatic activities and taxon-specific lethality between the Chinese and the Southeast Asian King Cobra venoms. The venom samples from Southeast Asia (Thailand, Malaysia, Indonesia) exhibited higher metalloproteinase, acetylcholinesterase and alkaline phosphatase activities than the Chinese samples. Taxon-specific lethality test was conducted in mice and lizards (*Eutropis multifasciata*) via intra-peritoneal route. It revealed that the Chinese samples were more lethal to mice with an *i.p.* LD_50_ of 0.5−0.6 µg/g, comparing to samples from Indonesia (1.1 µg/g), Malaysia (2.9 µg/g) and Thailand (3.5 µg/g), whereas the Southeast Asian samples were more lethal to lizards (Malaysia: 13.9 µg/g; Indonesia: 17.6 µg/g; Chinese/Guangxi: 30.2 µg/g; Thai and Hainanese samples not done) [21]. Another study compared the toxicity of three King Cobra venom samples, each derived from a single snake of Thai origin in different provinces (Phetchabun, Songkhla and Suratthani) [64], and found that the Phetchabun King Cobra venom was more cytotoxic and more lethal (however LD_50_ not reported) than the two other samples. Considering the very small sample number of this latter study, a conclusion from the geographic perspective could not be drawn as the variation observed is likely inter-individual difference.

**Figure 7.**
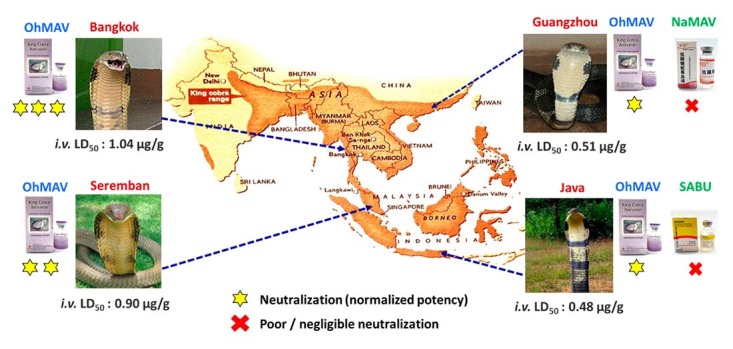
Schematic representation of geographical variation in the lethality of King Cobra venom sourced from different locales: Bangkok (Thailand), Seremban (West Malaysia), Guangzhou (China) and Java Island (Indonesia). The differential potency of King Cobra-specific antivenom, OhMAV and locally used non-specific antivenoms, NaMAV (China) and SABU (Indonesia), in neutralizing the venom lethality was illustrated. OhMAV: *Ophiophagus hannah* Monovalent Antivenom (produced in Thailand); NaMAV: *Naja atra* Monovalent Antivenom (produced in China); SABU: Serum Anti Bisa Ullar (produced in Indonesia).

More recently, the lethal activities of King Cobra venoms from four distant geographical locales (Thailand, Malaysia, Indonesia, and China) were shown to vary considerably [22]. The Chinese and Indonesian King Cobra venoms were the most lethal to mice (intravenous LD_50_ ~0.50 µg/g) in comparison to the Malaysian (LD_50_ = 0.90 µg/g) and Thai (LD_50_ = 1.04 µg/g) specimens. *In vivo* neutralization study revealed poor cross-neutralization of King Cobra venom lethalities by non-specific antivenoms, *i.e.*, the Chinese *Naja atra* Monovalent Antivenom (NaMAV) and Indonesian Serum Antibisa Ular (SABU), which are commonly used in the respective countries to treat King Cobra envenomation [22]. The species-specific antivenom, Thai OhMAV effectively neutralized the lethality of the King Cobra venoms to different extents. The study expressed the efficacy of the antivenom in median effect dose (ED_50_), defined as the volume dose of antivenom needed to protect half of the tested mice from venom-induced lethality. OhMAV was found more efficacious in neutralizing the venom of Thai origin (median effective dose, ED_50_ = 39.37 µL) than it was against the Malaysian (ED_50_ = 129.09 µL), Indonesian (ED_50_ = 139.58 µL) and Chinese (ED_50_ = 170.16 µL) venom samples. The observation strongly supports the species-specificity of OhMAV toward its immunogen of Thai origin, and underscores the impact of King Cobra venom variation on the effectiveness of antivenom treatment (Figure 7).

The existing data tend to support that the biochemistry and toxicity of King Cobra venom vary geographically, with a marked divergence between the Chinese and most Southeast Asian populations. From the ecological perspective, the variation in taxon-specific toxicity (in which the Chinese King Cobra venom was more lethal to mice while the Southeast Asian King Cobra venoms were more lethal to lizards) [21] suggests a possible dietary shift from mainly ophiophagy toward an enlarged scope of prey including mammals in the Chinese King Cobra, or inversely a dietary specialization from opportunistic feeding toward ophiophagy in the Southeast Asian King Cobra. The taxa-dependent toxicity of the venom is likely a result of arms races between King Cobra and available prey. Geographical differences in prey abundance, both ophidian and mammalian, between subtropical China (at a latitude above the tropic of Cancer, 23°26’) and tropical Southeast Asia, as well as anthropogenic factors such as habitat destruction or cultural practice (China being a well-known region historically suffering from pest rodents [88]), could have all contributed to the geographical variation in King Cobra venom, shaping divergent venom phenotype in the species.

## Antivenomics to decipher the limitation of antivenom neutralization

Antivenomics is applied to examine the immunorecognition of an antivenom to protein components in a venom. Through the identification of toxins that are poorly bound immunologically, the weakness of an antivenom can be identified for improvement. The information on King Cobra antivenomics is however limited. Using a 2DE-immunoblotting technique, Danpaiboon et al. [61] earlier investigated the immunological binding of Thai King Cobra venom by human single-chain variable antibody fragments specific to *N. kaouthia* LNTX (NkLN-HuScFv, produced in laboratory). The experimental hetero-specific antibody bound to a short and a long neurotoxin in King Cobra venom, while weakly cross-neutralized the lethality of the venom. Another study by Liu et al. [58] reported the cross-reactivity (applying 1DE-immunoblot) and cross-neutralization of Indonesian *O. hannah* venom by the Taiwanese neurotoxic bivalent antivenom (TNBAV, CDC product), which is raised against *Bungarus multicinctus* and *Naja atra* venoms. Similarly, the finding showed that TNBAV cross-immunoreacted with most *O. hannah* HPLC-eluted venom fractions but failed to cross-neutralize the venom toxicity in mice [58]. The phenomenon further supports the need to use a species-specific antivenom, *i.e.*, OhMAV, to achieve effective neutralization in King Cobra envenomation [22]. The differential efficacy of OhMAV against King Cobra venom of different locales, however, awaits elucidation with antivenomic approaches.

## Conclusions

King Cobra (*Ophiophagus hannah*) has a wide geographical distribution in Asia, and is a medically important venomous snake capable of causing fatal envenomation. Epidemiological data of King Cobra envenomation, as with that of snakebite in the region, is relatively scarce. Worldwide, the only species-specific antivenom indicated for treating King Cobra envenomation (*Ophiophagus hannah* Monovalent Antivenom, OhMAV) is produced in Thailand by the Queen Saovabha Memorial Institute, Bangkok, raised against the venom of Thai King Cobra. Clinical reports showed that high doses of the mono-specific King Cobra antivenom, often beyond 20 vials, were needed in the treatment of King Cobra envenomation. Preclinical assessment of OhMAV neutralization activity revealed variable efficacy against the venoms of King Cobra from different locales, indicating vast geographical variation in the composition and antigenicity of the venom. Geographical variation of the venom, however, has not been comprehensively investigated despite a number of proteomic studies reported on King Cobra venom. Discrepancies in the venom proteomes reported across the different studies were observed, presumably due to differences in the sampling and method of analysis, which resulted in varying depths of venom profiling and incompatible quantitation of protein abundances. Comparison of King Cobra venom proteomes reported from different studies for the interpretation of geographical venom variability thus remains challenging. Furthermore, mechanistic and antivenomic studies are also lacking to elucidate the clinical pathophysiology of envenomation and the variable effectiveness of OhMAV in neutralizing King Cobra venom. 

Future works should be tailored toward a comprehensive proteomic study of King Cobra venom from various locales, taking consideration into the potential variation arising from ontogenic factor and the environment of habitat (wild *v.s.* captivity). A standard method of proteomic analysis should be adopted, preferably one that incorporates the protein decomplexation strategy and high-resolution mass spectrometry coupled with deep database mining for better detection of protein diversity. The quantitation of protein should also be standardized, and in this context, the term “percentage” should be used cautiously to denote relative protein abundance, so that the composition of a protein is defined by its amount with respect to the total venom proteins. Furthermore, through high-performance liquid chromatography of the venom (protein decomplexation), it is possible to isolate the various venom proteins eluted for further functional investigation. This will help identify the key toxins and elucidate their toxicity, providing deeper insights into the pathophysiology and treatment of envenomation according to the variability of venom. Furthermore, the antivenomic approach based on either enzyme-link immunosorbent assay, immunoblotting or affinity chromatography, should follow to examine the degree of immunorecognition or immunocapturing capacity of OhMAV toward the different toxin components in the venom, for specimens originated from different geographical populations. Importantly, the immunologically-derived antivenomic findings must be interpreted against the *in vivo* efficacy of OhMAV, in which the venom and isolated toxin principles are subject to neutralization as outlined in functional antivenomics [65,89]. These are crucial to demonstrate the weakness or limitation of the antivenom in neutralizing the King Cobra venom of different geographical sources, so that the antivenom manufacturing can be improved to yield a product with higher potency and broader geographical utility. One promising approach is the potential use of fully human monoclonal antibodies as a recombinant antivenom targeting a particular animal toxin, for instance, dendrotoxins from the African Black Mamba [90]. Although recombinant antivenoms are possible alternatives to animal-derived antivenoms [91], the application probably remains far-reached in authors’ opinion. A simple, pragmatic and affordable approach can be taken to improve the existing antivenom production through a better process of antibody purification, and, by developing a pan-regional antivenom from immunogen mixture(s) containing the different target toxins in a formula optimized for hyperimmunization in host animals [92,93]. This shall contribute toward better management of snakebite envenomation in the region, in line with the WHO’s strategy to halve the disease burden of snakebite by 2030.
